# Factor structure of academic resilience among Polish and Ukrainian students involved in remote education caused by Covid-19 and military aggression

**DOI:** 10.1038/s41598-024-51388-x

**Published:** 2024-01-10

**Authors:** Tetiana Matusevych, Nataliia Demeshkant, Sławomir Trusz

**Affiliations:** 1 UNESCO Chair on Science Education, Dragomanov Ukrainian State University, Pyrohova 9, Kyiv, 01601 Ukraine; 2 Institute of Pedagogy, University of the National Education Commission, 4 Ingardena St., 30-060 Krakow, Poland; 3 Department of Pedagogy, WSB University, Cieplaka 1C St., 41-300 Dąbrowa Górnicza, Poland

**Keywords:** Psychology, Health care

## Abstract

Academic resilience explains how students overcome various challenges or negative experiences that can hinder the learning process. The COVID pandemic as well as war conflicts might be significant factors affecting the structure of the academic resilience of students. This study attempted to assess the extent to which the Cassidy’s construct of resilience can be used to interpret the behavior of other—Polish and Ukrainian samples, under remote education caused by the COVID-19 pandemic and Russian military aggression against the Ukrainian civils. Second, the relationships between resilience and students' self-efficacy were estimated. To test the factor structure of the resilience exploratory and confirmatory factor analyses were conducted. Assumed structure reproduced to a greater extent among Polish (83.4% similarity) than in Ukrainian respondents (from 27 to 40%) and it was three or two factors for Polish and Ukrainian students, respectively. General self-efficacy positively correlated with resilience both among Polish and Ukrainian respondents confirming the concurrent validity of the scale. The discovered differences were explained by differences in the historical and sociocultural experiences of the two nations. If among Ukrainian students historical and social experiences actually lead to the formation of a pattern of Perseverance in Overcoming Problems, then in the factor analysis, this pattern should be reproduced in the form of a single factor. At the same time, experiences with negative emotions should give a second-factor Negative affect and emotional response. The results obtained confirmed this assumption.

Academic resilience is a significant factor related to students' ability to adapt to the university environment and helps them reduce the risk of stress. It involves students' enjoyment of meeting all academic requirements, enhancing their academic achievements, and facilitating effective coping strategies when they experience academic stress^1^. Academic resilience explains how students overcome various challenges or significant negative experiences that can hinder the learning process. This enables individuals to adapt effectively and successfully complete their academic responsibilities. Resilience can be explained as an ability and a process that allows an individual to develop positive adaptation despite challenges and adversities^2,3^.

Over the last few years, people have been experiencing difficult conditions on a large scale including the COVID pandemic as well as war conflicts in areas that have been living without armed conflicts. The recent COVID-19 pandemic has brought changes in various aspects of life, which have become new challenges for students^4^. Higher education students experience rates of depression and anxiety substantially higher than those found in the general population^5^. A great deal of resilience is needed by all students and educators to get through the pandemic and to adapt to the huge impact it is having on education^6^. Student involvement by the ability to survive and face academic challenges during the online learning process; also called academic resilience^1^.

Regarding war conflicts, we mean first of all the Russian invasion of Ukraine, which has triggered an enormous humanitarian crisis, and has inflicted, and continues to inflict, deep and enduring harm on human health^8^. One of the groups most heavily affected, including the greatest impacts on health and well-being, is young people. The psychological impacts of the Russian invasion—triggered by sheltering from bombardment, migrating from homes, having families separated, witnessing the destruction of communities, and suffering the death of family members and friends—are hugely destabilizing.

Armed conflict significantly damages a nation’s education sector. Such damage takes various forms, including both direct and indirect damages to all participants of the educational process. Upon the outbreak of war, all schools across Ukraine were immediately closed and classroom learning replaced with online instruction. The harmful impacts of such interruptions to the academic learning, students’ social development and wellbeing, were revealed by the lockdowns mandated in response to COVID-19^9^. These problems now likely to be repeated and exacerbated by war. When students maintain schools activities during times of ongoing violence, and the school provides a positive emotional and physical climate, students demonstrate greater resilience^10^. Studies with the undergraduate students during pandemic lockdown reported on psychological impact of quarantine with following disorders: confusion, fear, numbness^11^.

Resilience and self-efficacy are very important individual resources to cope with these difficult conditions. Resilience refers to an ability and a process that allows individuals to thrive in the face of adversity^12,13^. Self-efficacy aims at a broad and stable sense of personal competence to deal effectively with a variety of stressful situations. It might reflect a generalization across various domains of functioning in which people judge how efficacious they are^14^.

One of the unique and novel approach to the measurement of academic resilience in university students is multidimensional construct which was proposed by Cassidy (2016), based on students’ specific adaptive cognitive-affective and behavioral responses to academic adversity^15^. Cassidy's process-based construct applies to the unique challenges faced by students during the COVID-19 pandemic and military aggression because it reflects the conceptual areas of self-efficacy and self-regulation together with the range of attributes, characteristics and factors commonly associated with resilience: confidence (self-efficacy), commitment (persistence), coordination (planning), control (how hard work and effective strategies impact achievement) and composure (low anxiety). Cassidy’s model of resilience is based on protective factors such as perseverance, help-seeking, emotional response that helps mitigate risk and adversity caused by unprecedented challenges caused by the threat to life and health during the Covid pandemic and the war in Ukraine.

Given the theoretical assumptions and empirical evidence discussed above, in the presented study was made an attempt to answer the following research questions:

1. in what extent the solution proposed by Cassidi (2016), the author of the multidimensional construct measure of academic resilience analyzed on a sample of British students, is reproduced considering data from Polish and Ukrainian students in difficult situations caused by the COVID-19 pandemic and the Russian military aggression against Ukraine?, and
2. what are the relationship of students’ resilience with their self-efficacy?

## Methods

### Design

The aims were realized in the cross-sectional study in the following steps: (1) translation of the original Academic Resilience Scale-30 (ARS-30)^9^ and the General Academic Self-Efficacy Scale (GASE)^16^ into national Ukrainian and Polish languages, (2) completing subsamples—one Polish and two Ukrainian. Polish (P21) and the first Ukrainian (U21) subsamples included respondents who experienced remote education caused pandemic COVID-19. The second Ukrainian (U22) subsample contained students who were studied online during Russian war aggression in Ukraine, (3) measuring students' resilience and their self-efficacy using adapted tools, (4) analysis of the collected data and comparison of the results with those obtained by Cassidy (2016).

The instrument was translated into Ukrainian by two certified translators. Then, it was checked the consistency of meaning for the parallel versions of the questionnaires by English Studies students whose first language was Ukrainian. The Polish version was prepared using a similar procedure.

### Participants

The subsamples were organized in accordance with voluntary sampling scheme^17^. First, employees of Polish and Ukrainian universities were contacted and asked to provide information about the study and encourage their students to fill out survey questionnaires. Links to electronic versions of the tools with instructions have been provided to university employees. Data collection was anonymous in order to improve the validity of responses and lasted from March 2021 to June 2022. For P21 and U21, the following selection criteria were followed: the individual had to be a university student, participate in classes remotely and had to give written consent to participate in the study. Regarding U22, in addition to the aforementioned criteria, an additional consideration was factored in; specifically, the individual had to reside in an area impacted by Russian military aggression.

Finally, empirical material was obtained from 582 undergraduate university students (aged 18–20 years), of which P21 covered 259 individuals, while U21 and U22 included 105 and 218 participants, respectively. Descriptive statistics for the subsamples are summarized in supplementary Table 1.

### Measures

Students' resilience was measured using the ARS-30 in translated versions. The questionnaire consists of 30 items based on student responses to academic adversity. In the original, validation study the instrument consists of a three-factor structure: Perseverance (F1), Reflective and Adaptive Help-seeking (F2), Negative Affectivity, and Emotional Response (F3).

Participants were also asked to complete the GASE according to the procedure proposed by Cassidy (2016). Both ARS-30 and GASE use a Likert scale with a range of 1 (very inappropriate) to 7 (very appropriate).

### Data analysis

The data analysis methods applied by the authors were analogous to those used by Cassidy in the original paper. This made it possible to compare the results and assess the applicability of the construct for data taken from various populations.

To determine reliability and the factor validity of the ARS, its psychometric properties were analyzed. First, the internal consistency of the instrument was quantified. Then, to test factor structure of the instrument exploratory and confirmatory factor analyses were conducted. Moreover, descriptive statistical analyses were carried out. Data analysis in this study was performed using the IBM SPSS-28 and AMOS-28 software.

The procedure, objectives and research tools were approved by the Research Ethic Committee of the home University of the corresponding author. All methods were carried out in accordance with relevant guidelines and regulations. Informed consent was obtained from all students participated in research.

### Ethical approval

The procedure, objectives and research tools were approved by the Research Ethic Committee of the Dragomanov Ukrainian State University, Kyiv, Ukraine.

## Results

### Reliability of the measurements

The reliability of the resilience measurement for the entire tool was high and ranged from Cronbach Alphas 0.86 to 0.89. Slightly lower, though still satisfactory results were obtained for particular factors, ranging from Cronbach Alphas 0.70 to 0.84. Detailed data for the analyzed subsamples are provided two last lines of supplementary Tables 2–3. Similarly, the reliability of the GASE measurement was high and amounted Cronbach Alphas: 0.810, 0.849, 0.828 for P21, U21 and U22, respectively.

### Factor structure of resilience

#### Results of the exploratory factor analyses (EFA): three-factor solution

Statistical analysis were conducted using the maximum likelihood method of factor extraction. Supplementary Table 2 shows factor loadings after promax rotation. Item clustering by Cassidy (2016) suggests that F1 includes items 1, 2, 3, 4, 5, 8, 9, 10, 11, 13, 15, 16, 17, and 30; F2 contains items 18, 20, 21, 22, 24, 25, 26, 27, and 29 and F3 covers items 6, 7, 12, 14, 19, 23, and 28^15^.

Concerning Polish students, 83.4% of the items follow the solution proposed by Cassidi (2016). F1 included 10 from 14 items originally designated to this Factor (items #2, 3, 4, 5, 9, 10, 11, 15, 16, 17). Poles interpret perseverance similarly to original study, however there was also one item (#7) that according to Cassidy (2016) characterized F3^15^. This item allocation might indicate change in respondents' attitude towards previous life choices. Furthermore, item #24 originally assigned to F2 was moved to F1. Perhaps, this item for Polish students represented strategy of the perseverance applied by them in the difficult situation of remote learning during the COVID-19 pandemic. Other items originally characterized F1 (#8, 13, 30) moved to F2. The answers to these items were referred to reflecting and adaptive help-seeking strategy and represented a combination of cognitive-affective and behavioral responses. One item #1 originally belongs to F1 moved to F3. This shift can be explained by the emotional perception of its content.

Regarding F2, it contained 8 items (#18, 20, 21, 22, 25, 26, 27, 29) out of 9 identified by Cassidy (2016)^15^. The result indicated almost complete agreement in the interpretation of this factor by Polish respondents with those in the original study. It may indicate similarities in the structure of academic resilience of Polish students with their peers from Western European countries. Furthermore, there were 3 items (#8, 13, 30) characterizing F1. This shift may represent respondents’ perception of these items not as referring to perseverance, but as the strategy of seeking help in a stressful situation.

Concerning F3, it is necessary to emphasize nearly full correspondence of the obtained results with Cassidy (2016) findings. F3 included six items (#6, 12, 14, 18, 23, 28) out of seven originally identified. In general, Polish students interpreted F3 similarly to British respondents as a factor related to affect associated with problem situations and catastrophic thinking.

Considering Ukrainian students during pandemic Covid in 2021, 40% of the items follow the solution proposed by Cassidi (2016). F1 included items which originally pertained to F1 (#2, 3, 4, 8, 11, 13, 15, 16, 30) and F2 (#18, 20, 21, 22, 25, 26, 27, 29). Furthermore, F2 partially took items from F1 *(*#5, 9, 10, 17*)* and F3 (#7, 12, 19, 23, 28). The result obtained is noticeably different from the Cassidy (2016) concept as well as from the factor structure recorded among P21.

Regarding F3, out of 7 items originally assigned to F3, only two items (approximately 28%) were reproduced in U21. It seems that the Cassidy's proposal to isolate a 3-factor structure with negative mood seems as the last factor is inadequate for Ukrainian students.

Analyzing the findings obtained for U22, only 27% of the items reproduced the factor structure proposed by Cassidy (2016). F1 was loaded by items originally associated with F1 (#2, 3, 8, 9, 11, 13, 16, 30) and F2 (#18, 20, 22, 24, 25, 26, 27, 29). Moreover, F2 did not include any original item but it covered some items both from F3 (#6, 7, 12, 14, 19, 23, 28) and F1 *(*#5, 10, 15).

Finally, F3 was loaded by 3 items originally related to F1 (#1, 4, 17) and one item (#21) from F2. The results of U21 and U22 allow to assume that F1 representing perseverance captured the items of F2 corresponding to help-seeking*.* In other words, the EFA results showed specifics of Ukrainian participants' approach to the perseverance as a resilience component. In their view perseverance is perceived as an interpretation of the events combined with action.

#### Results of the EFA: two-factor solution

Considering the findings obtained for U21 and U22 i inconsistent with postulated by Cassidy (2016), a two-factor resilience structure was proposed. It was assumed that the new F1 will be loaded by the items originally assigned to Perseverance and Reflecting and Adaptive Help-Seeking factors, whereas the new F2 will capture the items previously related to Negative affect and emotional response factor.

The two-factor structure was tested in consecutive EFAs. The findings are presented in supplementary Table 3.

As presumed, the new F1 described Ukrainian students' experience of motivating themselves, putting more effort to achieve goals, treating failures as challenges, monitoring their own actions, seeking support from significant others etc. The new F1 for U21 was loaded by 11 items originally tied to Perseverance factor (#2, 3, 4, 8, 9, 11, 13, 15, 16, 17, 30) and 8 items associated with Reflecting and Adaptive Help-Seeking factor (#18, 20, 21, 22, 24, 25, 27, 29). Therefore, the discussed factor was named *Perseverance in Overcoming Problems*.

The new F2 for U21 contained items describing negative emotions resulting from failures and depressive anticipation of lack of success in school and work life. Thus, the new F2 was named Negative affect and emotional response analogous to the original F3. This factor covered 10 items, of which 7 (#6, 7, 12, 14, 19, 23, 28) loaded the original F3. The three other items originated from F1, but the factor loading of item #1 was negligible, while the relations of item #5 and #10, due to their contents, are difficult to interpret clearly and sensibly.

The EFA results for U22 were similar to above described for U21. The new F1 (*Perseverance in Overcoming Problems)* was loaded also by 19 items, of which ten (#2, 3, 4, 8, 9, 11, 13, 16, 17, 30) correlated with the original F1, whereas nine (#18, 20, 21, 22, 24, 25, 26, 27, 29) originated from F2. Similarly for the new F2 (*Negative Affect and Emotional Response)* it was loaded by seven items (#6, 7, 12, 14, 19, 23, 28) correlated with original F3. The four other items either correlated low with the new F2 (#1) or their relationships with the factor were difficult to interpret (#5, 10, 15).

#### Results of the confirmatory factor analyses (CFA)

To ascertain to what extent the 3 or 2-factor solutions are adequate, the CFAs were carried out for particular subsamples. The results obtained are presented in supplementary Tables 4–6.

The results obtained for P21 show that the proposed model matched the data well: χ^2^(306) = 1.32; p = 0.056; RMSEA = 0.23; 90% CI = 0.00 – 0.034; CFI = 0.988, TLI = 0.984, NFI = 0.912, AGFI = 0.878). The ARS items were significantly and strongly linked to the extracted factors: for F1, F2, and F3 averaged values of *βs* were 0.605, 0.528, and 0.629 respectively.

The hypothesized two-factor models for Ukrainian students were also fitted well to the data (for U21: χ^2^(349) = 1.119, p = 0.062, RMSEA = 0.034; 90% CI = 0.00–0.051, CFI = 0.970, TLI = 0.962, NFI = 0.783, AGFI = 0.764 and for U22: χ^2^(319) = 1.129, p = 0.055, RMSEA = 0.24; 90% CI = 0.00–0.036, CFI = 0.982, TLI = 0.975, NFI = 0.867, AGFI = 0.860). Again, the ARS items correlated with the extracted factors. Averaged values of *βs* for F1 and F2 were 0.518 and 0.637 (U21), and 0.488 and 0.569 (U22).

### The relationship of resilience to academic self-efficacy

Considering the three-factor solution for all subsamples, the correlations between GASE and ARS30 were positive, and their power was medium except F3 among U21, where this link was weaker (see Table 1). For the two-factor solution in U21 and U22, the correlations between ARS30 and GASE also were positive with a medium power (see Table 2). This means that as the level of general self-efficacy increased, the level of resilience in general terms and in relation to the identified dimensions also improved.

**Table 1:** Correlation coefficients between ARS-30 and general academic self-efficacy scale (GASE) for 3 factors solution.

ARS 30	GASE		
	P21 (N = 259)	U21 (N = 105)	U22 (*N* = 218)
Global score	.647**	.572**	.573**
F1	.641**	.412**	.473**
F2	.483**	.523**	.433**
F3	.473**	.240*	.420**

**Table 2:** Correlation coefficients between ARS-30 and General Academic Self-Efficacy Scale (GASE) for 2 factors solution.

ARS 30	GASE	
	U21 (N = 105)	U22 (*N* = 218)
Global score	.572**	.573**
F1	.462**	.508**
F2	.479**	.468**

## Discussion and conclusions

The study pursued two objectives. First, an attempt was made to assess to what extent the multidimensional resilience measurement tool proposed by Cassidy (2016) can be used to analyze this trait among Polish and Ukrainian students. In general, we found that the resilience structure postulated by Cassidy reproduced to a greater extent in P21 (83.4% similarity) than in U21 (40%) and U22 (27%).

In P21 the three-factor solution was obtained, while in U21 and U22, it was two-factor. For P21 factor 1 was interpreted as *perseverance*, includes items featuring hard work and trying, not giving up, sticking to plans and goals, accepting and utilizing feedback, imaginative problem solving and treating adversity as an opportunity to meet challenges and improve as central themes^15^. Over the past thirty years, a number of studies have justified structure perseverance. Namely, willingness to continue to struggle and to practice self-discipline^18^, personal control and tenacity^19^, hard work and effective strategies^20^, and personal control and goal orientation^21^.

Items loading on factor 2, *reflecting and adaptive-help-seeking*, features themes including reflecting on strengths and weakness, altering approaches to study, seeking help, support and encouragement, monitoring effort and achievements and administering reward and punishments^15^. This factor contains items related to belief in one’s capabilities and recognizing personal strengths and limitations^22^, adaptability^21^ and adaptive help-seeking^23^.

Finally, factor 3, *negative affect and emotional response features* themes including anxiety, catastrophizing, avoiding negative emotional responses, optimism, and hopelessness. The structure of this factor is similar to acceptance of negative affect^19,21^, composure^20^, and meaningfulness^18^.

Among U21 and U22, a two-factor solution was obtained in both EFA and CFA analysis. Ukrainian students were characterized by a specific approach to perseverance as resilience component. This was perceived as an interpretation of the events combined with action. Interpreting the resilience as an action-oriented process emphasizes the modifiable properties rather than the fixed conditions of challenging situations that student veterans face. Consequently, military veterans are able to initiate necessary changes to achieve a better life^3^. Stressful situations, such as armed conflicts, appear to serve some people as an opportunity for revealing useful coping strategies and resilience^24,25^.

Changes in the structure of the resilience can be explained by the historical and cultural background of Ukraine as a state. The Ukrainian historical context is associated with permanent efforts and even fighting for their own (including independence). This might affect the mentality of the Ukrainian people, especially in the interpretation of perseverance^26,27^. In this way, perseverance transforms into permanent action (persevering overcoming problems). In other words, is was observed a cultural based mixing of the factor 1 (*Perseverance*) with factor 2 (*Reflecting and Adaptive Help-Seeking*). This creates a qualitatively new dimension—*Perseverance in Overcoming Problems*.

The structural alteration of the resilience construct can also be explained within Folkman and Lazarus (1988) concept of stress coping styles. They distinguished three strategies and one of which is a problem strategy. Authors proposed four types of coping which are strongly associated with changes in emotion: planful problem-solving, positive reappraisal, confrontive coping, and distancing^28^.

According to their study planful problem-solving was associated with an improved emotion state; it was associated with less negative emotion and more positive emotion. It cannot be ruled out that people can begin to feel better when they turn to the problem that is causing distress. Another explanation is that planful problem-solving, when effective, can result in an improved person-environment relationship, which should in turn lead to a more favorable cognitive appraisal and hence a more positive emotion response^28–30^.

As for the relationship between resilience and cultural background, similar conclusions are proposed by Bogdanov et al. (2021). These authors point to the need for contextual, culturally relevant measures of resilience for war-affected adolescents in Eastern Ukraine what is in short supply in Eastern Europe^31^. The authors point out that in the case of Ukrainian adolescents, the process of cultural adaptation as well as strength and difficulties as the resilience components should be taken into account^32,33^. In the research of Bogdanov et al. (2021) uses measure, which has a three-factor structure—individual, relational, and contextual^34^, includes a local functioning scale that offers the possibility of contextualizing it to specific cultures and environments.

It was assumed that if historical and social experiences in the group of Ukrainian students actually lead to the formation of a pattern of *Perseverance in Overcoming Problems*, then in the factor analysis this pattern should be reproduced in the form of a single factor. At the same time, experiences about negative emotions should give a second factor *Negative affect and emotional response*. The results obtained confirmed this assumption.

The second objective of the study was to estimate the relationship between resilience and students self-efficacy. GASE positively correlated with resilience in both Polish and Ukrainian respondents, confirming the concurrent validity of the scale. Research suggests that self-efficacy is an important contributory factor for resilience^15,20,35^. Self-efficacy can build academic resilience, and on the other hand, resiliency can enhance self-efficacy. The result obtained is corresponding to that reported by Cassidy (2016) and other authors analyzing the relationship between these two constructs^22,36,37^.

Obtained results develop resilience theory proposed by Cassidy. They make the construct can be used in various populations. Moreover, they provide an impetus for further research in which the structure of resilience will be modified taking into account the specificity of the respondents' experiences, especially in difficult life situations, the solution of which requires the resources postulated by Cassidy.

On the other hand, discussed findings have great practical value. An accurate diagnosis of resilience allows for the design of intervention programs with empirically confirmed effectiveness, as opposed to random or speculative, commonsense, anecdotal approaches.

This study has some limitations. Three of them seem to be the most relevant. First, using in this study the resilience scale proposed by Cassidy (2016), the measurement was conducted according to a slightly different procedure compared to the original one. Participants in this study were diagnosed in natural situations (the COVID-19 pandemic and military conflict in Ukraine). In contrast, Cassidy (2016) measured resilience in a quasi-experimental procedure, previously presenting respondents with two independent versions of the academic adversity vignette.

The second limitation is characteristic of cross-sectional surveys. The measurement was conducted once and the results obtained could be to some extent random, resulting from the influence of various uncontrolled contextual variables. For example, the group of such variables may include temperament, personality traits that influence people's resistance to various types of stressors, including the threat of disease or aggression from others^38^. It was not ruled out that different results could have been obtained in longitudinal studies, which would track the development of resilience in changing circumstances. The power of conclusions in this type of research would increase for randomized trials^39^.

Third, the comparison groups in this study were not equal and participants were involved using not random but volunteer sampling scheme. Therefore, it cannot be ruled out that other factors motivated Ukrainian and other Polish respondents to participate in the survey. Ultimately, this may have affected the findings.

### Supplementary Information

Supplementary Information.

## Data Availability

The datasets used and/or analysed during the current study available from the corresponding author on reasonable request.
