# Supplementary material for: Factor structure of academic resilience among Polish and Ukrainian students involved in remote education caused by Covid-19 and military aggression
Source: Sci Rep. 2024 Jan 10;14:1000. doi: 10.1038/s41598-024-51388-x (PMC10781683; doi:10.1038/s41598-024-51388-x)
Supplement: Supplementary file 1 — Supplementary Information. [file 41598_2024_51388_MOESM1_ESM.docx]

**Supplementary Table 1.** Details on demographics of study subsamples

| Subsamples | *N* | Male | Female | others | Mean Age SD |
| --- | --- | --- | --- | --- | --- |
| P21 | 259 | 31 | 223 | 5 | 19.90 (1.42) |
| U21 | 105 | 69 | 31 | 5 | 17.97 (0.18) |
| U22 | 218 | 108 | 103 | 7 | 18.65 (0.97) |
| Total | 582 | 208 | 357 | 17 | 18.84 (0.85) |

**Supplementary Table 2.** The EFA results for the resilience among P21, U21 and U22 subsamples

| ARS-30 Item | | Factor loadings | | | | | | | | |
| --- | --- | --- | --- | --- | --- | --- | --- | --- | --- | --- |
|  |  | Subsample | | | | | | | | |
|  |  | P21 | | | U21 | | | U22 | | |
|  |  | F1 | F2 | F3 | F1 | F2 | F3 | F1 | F2 | F3 |
| 1 | I would not accept the tutors’ feedback |  |  | 0.041 |  |  | 0.065 |  |  | 0.123 |
| 2 | I would use the feedback to improve my work | **0.367** |  |  | **0.370** |  |  | **0.450** |  |  |
| 3 | I would just give up | **0.720** |  |  | **0.534** |  |  | **0.570** |  |  |
| 4 | I would use the situation to motivate myself | **0.704** |  |  | **0.754** |  |  |  |  | 0.875 |
| 5 | I would change my career plans | **0.658** |  |  |  | 0.527 |  |  | 0.403 |  |
| 6 | I would probably get annoyed |  |  | **0.222** |  |  | **0.499** |  | 0.360 |  |
| 7 | I would begin to think my chances of success at university were poor | 0.594 |  |  |  | 0.699 |  |  | 0.720 |  |
| 8 | I would see the situation as a challenge |  | 0.505 |  | **0.576** |  |  | **0.451** |  |  |
| 9 | I would do my best to stop thinking negative thoughts | **0.611** |  |  |  | 0.512 |  | **0.546** |  |  |
| 10 | I would see the situation as temporary | **0.691** |  |  |  | 0.721 |  |  | 0.332 |  |
| 11 | I would work harder | **0.566** |  |  | **0.664** |  |  | **0.642** |  |  |
| 12 | I would probably get depressed |  |  | **0.715** |  | 0.645 |  |  | 0.733 |  |
| 13 | I would try to think of new solutions |  | 0.612 |  | **0.443** |  |  | **0.447** |  |  |
| 14 | I would be very disappointed |  |  | **0.458** |  |  | **0.672** |  | 0.568 |  |
| 15 | I would blame the tutor | **0.169** |  |  | **0.349** |  |  |  | 0.435 |  |
| 16 | I would keep trying | **0.721** |  |  | **0.664** |  |  | **0.643** |  |  |
| 17 | I would not change my long-term goals and ambitions | **0.611** |  |  |  | 0.583 |  |  |  | 0.398 |
| 18 | I would use my past successes to help motivate myself |  | **0.632** |  | 0.466 |  |  | 0.583 |  |  |
| 19 | I would begin to think my chances of getting the job I want were poor |  |  | **0.637** |  | 0.612 |  |  | 0.596 |  |
| 20 | I would start to monitor and evaluate my achievements and effort |  | **0.704** |  | 0.591 |  |  | 0.483 |  |  |
| 21 | I would seek help from my tutors |  | **0.430** |  | 0.557 |  |  |  |  | 0.434 |
| 22 | I would give myself encouragement |  | **0.602** |  | 0.820 |  |  | 0.692 |  |  |
| 23 | I would stop myself from panicking |  |  | **0.688** |  | 0.745 |  |  | 0.574 |  |
| 24 | I would try different ways to study | 0.566 |  |  |  | **0.371** |  | 0.438 |  |  |
| 25 | I would set my own goals for achievement |  | **0.655** |  | 0.603 |  |  | 0.647 |  |  |
| 26 | I would seek encouragement from my family and friends |  | **0.448** |  | 0.006 |  |  | 0.255 |  |  |
| 27 | I would try to think more about my strengths and weaknesses to help me work better |  | **0.674** |  | 0.684 |  |  | 0,741 |  |  |
| 28 | I would feel like everything was ruined and was going wrong |  |  | **0.745** |  | 0.687 |  |  | 0.663 |  |
| 29 | I would start to self-impose rewards and punishments depending on my performance |  | **0.486** |  | 0.138 |  |  | 0.157 |  |  |
| 30 | I would look forward to showing that I can improve my grades |  | 0.421 |  | **0.398** |  |  | **0.420** |  |  |
|  | **KMO**  0.9 | **Bartlett’s sphericity**  Χ^2^3133.735 (435) | |  | **Bartlett’s sphericity**  Χ^2^ 1383.607 (435) | |  | **Bartlett’s sphericity**  Χ^2^2316.689 (435) | |  |
|  | **Eigenvalues** | 8.782 | 2.903 | 1.824 | 8.072 | 3.263 | 1.9 | 7.204 | 3.279 | 1.777 |
|  | **% of variance** | 29.272 | 9.677 | 6.08 | 26.906 | 10.878 | 6.333 | 24.014 | 10.929 | 5.925 |
|  | **Cronbach’s α** | 0.824 | 0.805 | 0.795 | 0.777 | 0.700 | 0.841 | 0.741 | 0.707 | 0.805 |
|  | **Cronbach’s α for all items** | 0.89 | | | 0.87 | | | 0.86 | | |

Note: F1 – *Perseverance*; F2 - *Reflecting and Adaptive Help-Seeking*; F3 - *Negative affect and emotional response.*

Items factor loadings corresponding to the solution proposed by Cassidy (2016) are in bold

**Supplementary Table 3**. The EFA results for the resilience among U21 and U22 subsamples (two-factor solution)

| **ARS-30 Item** | | Factor loadings | | | |
| --- | --- | --- | --- | --- | --- |
|  |  | Subsamples | | | |
|  |  | U21 | | U22 | |
|  |  | F1 | F2 | F1 | F2 |
| 1 | I would not accept the tutors’ feedback |  | 0,062 |  | 0,139 |
| 2 | I would use the feedback to improve my work | **0,381** |  | **0,487** |  |
| 3 | I would just give up | **0,558** |  | **0,627** |  |
| 4 | I would use the situation to motivate myself | **0,796** |  | **0,731** |  |
| 5 | I would change my career plans |  | 0,472 |  | 0,443 |
| 6 | I would probably get annoyed |  | **0,264** |  | **0,342** |
| 7 | I would begin to think my chances of success at university were poor |  | **0,727** |  | **0,756** |
| 8 | I would see the situation as a challenge | **0,557** |  | **0,477** |  |
| 9 | I would do my best to stop thinking negative thoughts | **0,533** |  | **0,569** |  |
| 10 | I would see the situation as temporary |  | 0,586 |  | 0,361 |
| 11 | I would work harder | **0,679** |  | **0,662** |  |
| 12 | I would probably get depressed |  | **0,740** |  | **0,719** |
| 13 | I would try to think of new solutions | **0,467** |  | **0,484** |  |
| 14 | I would be very disappointed |  | **0,496** |  | **0,554** |
| 15 | I would blame the tutor | **0,288** |  |  | 0,447 |
| 16 | I would keep trying | **0,662** |  | **0,704** |  |
| 17 | I would not change my long-term goals and ambitions | **0,494** |  | **0,213** |  |
| 18 | I would use my past successes to help motivate myself | **0,501** |  | **0,546** |  |
| 19 | I would begin to think my chances of getting the job I want were poor |  | **0,675** |  | **0,621** |
| 20 | I would start to monitor and evaluate my achievements and effort | **0,516** |  | **0,443** |  |
| 21 | I would seek help from my tutors | **0,501** |  | **0,451** |  |
| 22 | I would give myself encouragement | **0,827** |  | **0,666** |  |
| 23 | I would stop myself from panicking |  | **0,797** |  | **0,545** |
| 24 | I would try different ways to study | **0,412** |  | **0,416** |  |
| 25 | I would set my own goals for achievement | **0,647** |  | **0,585** |  |
| 26 | I would seek encouragement from my family and friends |  | -0,302 | **0,226** |  |
| 27 | I would try to think more about my strengths and weaknesses to help me work better | **0,694** |  | **0,655** |  |
| 28 | I would feel like everything was ruined and was going wrong |  | **0,791** |  | **0,680** |
| 29 | I would start to self-impose rewards and punishments depending on my performance | **0,129** |  | **0,103** |  |
| 30 | I would look forward to showing that I can improve my grades | **0,379** |  | **0,438** |  |
|  | **KMO**  0,815 | **Bartlett’s sphericity**  Χ^2^1383,607 (435) | | **Bartlett’s sphericity**  Χ^2^2316,689 (435) | |
|  | **Eigenvalues** | 8,072 | 3,263 | 7,204 | 3,279 |
|  | **% of variance** | 26,906 | 10,878 | 24,014 | 10,929 |
|  | **Cronbach’s α** | 0,841 | 0,841 | 0,824 | 0,805 |
|  | **Cronbach’s α for all items** | 0,866 | | 0,855 | |

Note: F1 – *Perseverance in Overcoming Problems*; F2 - *Negative Affect and Emotional Response.*

Items factor loadings corresponding to the solution proposed by Cassidy (2016) are in bold

**Supplementary Table 4.** Standardized regression weights for P21

| ARS |  | Factor | Beta | b | S.E. | C.R. | p |
| --- | --- | --- | --- | --- | --- | --- | --- |
| ARS1 | <--- | F1 | -.003 | -.007 | .153 | -0.046 | .964 |
| ARS2 | <--- | F1 | .371 | .514 | .093 | 5.506 | ***  ***  ***  ***  *** |
| ARS3 | <--- | F1 | .689 | 1.330 | .142 | 9.388 |  |
| ARS4 | <--- | F1 | .984 | .983 | .085 | 11.618 |  |
| ARS5 | <--- | F1 | .572 | 1.007 | .122 | 8.279 |  |
| ARS8 | <--- | F1 | .603 | 1.413 | .165 | 8.563 |  |
| ARS9 | <--- | F1 | .685 | 1.589 | .168 | 9.439 | ***  ***  ***  ***  ***  ***  ***  *** |
| ARS10 | <--- | F1 | .720 | 1.445 | .163 | 8.882 |  |
| ARS11 | <--- | F1 | .577 | 1.947 | .354 | 5.506 |  |
| ARS13 | <--- | F1 | .628 | 1.086 | .123 | 8.836 |  |
| ARS15 | <--- | F1 | .215 | .445 | .134 | 3.322 |  |
| ARS16 | <--- | F1 | .741 | 1.129 | .114 | 9.866 |  |
| ARS17 | <--- | F1 | .584 | 1.355 | .176 | 7.713 |  |
| ARS30 | <--- | F1 | .500 | 1.106 | .151 | 7.339 |  |
| ARS18 | <--- | F2 | .756 | 1.329 | .165 | 8.066 | ***  ***  ***  *** |
| ARS20 | <--- | F2 | .518 | .880 | .116 | 7.592 |  |
| ARS21 | <--- | F2 | .389 | .813 | .147 | 5.526 |  |
| ARS22 | <--- | F2 | .600 | .882 | .122 | 7.252 |  |
| ARS24 | <--- | F2 | .600 | .812 | .115 | 7.038 | ***  ***  *** |
| ARS25 | <--- | F2 | .706 | .991 | .119 | 8.362 |  |
| ARS26 | <--- | F2 | .345 | .714 | .133 | 5.353 |  |
| ARS27 | <--- | F2 | .534 | .752 | .093 | 8.066 | *** |
| ARS29 | <--- | F2 | .303 | .635 | .138 | 4.608 | *** |
| ARS6 | <--- | F3 | .129 | .118 | .059 | 2.002 | .045 |
| ARS7 | <--- | F3 | .704 | .896 | .086 | 10.369 | *** |
| ARS12 | <--- | F3 | .693 | .871 | .080 | 10.834 | *** |
| ARS14 | <--- | F3 | .237 | .236 | .063 | 3.758 | *** |
| ARS19 | <--- | F3 | .744 | .843 | .073 | 11.598 | *** |
| ARS23 | <--- | F3 | .664 | .856 | .089 | 9.606 | *** |
| ARS28 | <--- | F3 | .735 | 1.116 | .108 | 10.369 | *** |

Note: * p< .05; ** p< .01; ***p< .001, S.E. - Standard error of regression weight, C.R. - Critical ratio for regression weight

**Supplementary Table 5**. Standardized regression weights for U21

| ARS |  | Factor | Beta | b | S.E. | C.R. | p |
| --- | --- | --- | --- | --- | --- | --- | --- |
| ARS1 | <--- | F1 | .095 | .194 | .208 | .936 | .349 |
| ARS2 | <--- | F1 | .457 | .779 | .174 | 4.472 | *** |
| ARS3 | <--- | F1 | .606 | .975 | .169 | 5.755 | ***  ***  ***  ***  ***  *** |
| ARS4 | <--- | F1 | .998 | .825 | .100 | 8.261 |  |
| ARS5 | <--- | F1 | .852 | 1.330 | .201 | 4.232 |  |
| ARS8 | <--- | F1 | .615 | 1.213 | .240 | 5.064 |  |
| ARS9 | <--- | F1 | .612 | 1.182 | .202 | 5.856 |  |
| ARS10 | <--- | F1 | .602 | 1.413 | .168 | 5.770 |  |
| ARS11 | <--- | F1 | .624 | 1.151 | .190 | 6.057 | ***  ***  .004  ***  ***  ***  ***  *** |
| ARS13 | <--- | F1 | .452 | .707 | .159 | 4.458 |  |
| ARS15 | <--- | F1 | .283 | .579 | .201 | 2.882 |  |
| ARS16 | <--- | F1 | .640 | .869 | .143 | 6.057 |  |
| ARS17 | <--- | F1 | .619 | 1.277 | .218 | 5.853 |  |
| ARS18 | <--- | F1 | .386 | .610 | .157 | 3.879 |  |
| ARS20 | <--- | F1 | .410 | .707 | .173 | 4.098 |  |
| ARS21 | <--- | F1 | .315 | .632 | .178 | 3.553 |  |
| ARS22 | <--- | F1 | .676 | 1.009 | .143 | 7.039 | ***  .001  ***  .392  *** |
| ARS24 | <--- | F1 | .320 | .441 | .136 | 3.256 |  |
| ARS25 | <--- | F1 | .496 | .701 | .144 | 4.875 |  |
| ARS26 | <--- | F1 | -.079 | -.17 | .198 | -.856 |  |
| ARS27 | <--- | F1 | .571 | .831 | .131 | 6.323 |  |
| ARS29 | <--- | F1 | .040 | .088 | .206 | .429 | .668 |
| ARS30 | <--- | F1 | .264 | .515 | .197 | 2.613 | .009 |
| ARS6 | <--- | F2 | .308 | .386 | .122 | 3.161 | .002 |
| ARS7 | <--- | F2 | .774 | 1.073 | .134 | 8.003 | *** |
| ARS12 | <--- | F2 | .745 | 1.018 | .126 | 8.071 | *** |
| ARS14 | <--- | F2 | .461 | .593 | .123 | 4.828 | *** |
| ARS19 | <--- | F2 | .644 | .761 | .112 | 6.803 | *** |
| ARS23 | <--- | F2 | .771 | .963 | .118 | 8.164 | *** |
| ARS28 | <--- | F2 | .756 | .932 | .116 | 8.003 | *** |

* p< .05; ** p< .01; ***p< .001, S.E. - Standard error of regression weight, C.R. - Critical ratio for regression weight

**Supplementary Table 6**. Standardized regression weights for U22

| ARS |  | Factor | Beta | b | S.E. | C.R. | p |
| --- | --- | --- | --- | --- | --- | --- | --- |
| ARS1 | <--- | F1 | -0.013 | -.024 | .134 | -.178 | .859 |
| ARS2 | <--- | F1 | .507 | .825 | .111 | 7.429 | *** |
| ARS3 | <--- | F1 | .594 | 1.050 | .123 | 8.558 | ***  ***  ***  ***  ***  *** |
| ARS4 | <--- | F1 | 1.005 | .728 | .062 | 11.703 |  |
| ARS5 | <--- | F1 | .276 | .556 | .142 | 3.926 |  |
| ARS8 | <--- | F1 | .479 | .949 | .133 | 7.146 |  |
| ARS9 | <--- | F1 | .636 | 1.083 | .119 | 9.066 |  |
| ARS10 | <--- | F1 | .365 | .551 | .099 | 5.555 |  |
| ARS11 | <--- | F1 | .615 | 1.057 | .113 | 9.354 | ***  ***  ***  ***  .013  ***  ***  *** |
| ARS13 | <--- | F1 | .457 | .662 | .097 | 6.823 |  |
| ARS15 | <--- | F1 | .344 | .612 | .113 | 5.434 |  |
| ARS16 | <--- | F1 | .616 | .946 | .101 | 9.354 |  |
| ARS17 | <--- | F1 | .175 | .350 | .142 | 2.471 |  |
| ARS18 | <--- | F1 | .510 | .864 | .129 | 6.718 |  |
| ARS20 | <--- | F1 | .345 | .596 | .112 | 5.318 |  |
| ARS21 | <--- | F1 | .351 | .540 | .101 | 5.356 |  |
| ARS22 | <--- | F1 | .722 | 1.150 | .116 | 9.895 | ***  ***  ***  .014  *** |
| ARS24 | <--- | F1 | .378 | .558 | .097 | 5.778 |  |
| ARS25 | <--- | F1 | .630 | .918 | .114 | 8.084 |  |
| ARS26 | <--- | F1 | .180 | .366 | .149 | 2.460 |  |
| ARS27 | <--- | F1 | .650 | 1.062 | .128 | 8.295 |  |
| ARS29 | <--- | F1 | .101 | .211 | .130 | 1.627 | .104 |
| ARS30 | <--- | F1 | .426 | .794 | .124 | 6.397 | *** |
| ARS6 | <--- | F2 | .285 | .364 | .093 | 3.913 | *** |
| ARS7 | <--- | F2 | .678 | .908 | .096 | 9.498 | *** |
| ARS12 | <--- | F2 | .730 | .889 | .092 | 9.711 | *** |
| ARS14 | <--- | F2 | .480 | .640 | .097 | 6.612 | *** |
| ARS19 | <--- | F2 | .492 | .589 | .087 | 6.807 | *** |
| ARS23 | <--- | F2 | .586 | .736 | .090 | 8.213 | *** |
| ARS28 | <--- | F2 | .735 | 1.102 | .116 | 9.498 | *** |

* p< .05; ** p< .01; ***p< .001, S.E. - Standard error of regression weight, C.R. - Critical ratio for regression weight
